# Strong Field Enhancement and Unidirectional Scattering Based on Asymmetric Nanoantenna

**DOI:** 10.3390/nano12122084

**Published:** 2022-06-16

**Authors:** Dengchao Huang, Shilin Liu, Wei Li, Kang Yang, Ting Peng

**Affiliations:** 1 Key Ministry of Education Laboratory of Advanced Perception and Intelligent Control of High-End Equipment, College of Electrical Engineering, Anhui Polytechnic University, Wuhu 241000, China; huangdengchao19@163.com (D.H.); sl.liu@ahpu.edu.cn (S.L.); liwei@ahpu.edu.cn (W.L.); 2 School of Electronic Information Engineering, China West Normal University, Nanchong 637001, China; ting653028@163.com

**Keywords:** hybrid nanoantenna, unidirectional emission, local field enhancement

## Abstract

Dielectric-metal nanostructures have lately emerged as one of the most promising approaches to modulating light at the optical frequency. Their remarkable electric and magnetic resonances give them a one-of-a-kind ability to augment local field enhancements with negligible absorption losses. Here, we propose a hybrid metal-dielectric-metal (MDM) nanoantenna that contains a dimer of three-layers of shell nanoparticles. In addition, we only theoretically and numerically show the optical properties of the hybrid dimer nanoantenna. We found that the nanoantenna sustained unidirectional forward scattering with narrow beamwidth (30.9 deg) and strong scattering intensity (up to 5 times larger than the single MDM particle). Furthermore, when the hybrid asymmetric dimer was excited by the plane wave with different electric polarization directions, our findings revealed that the hybrid nanoantenna boosted the gap’s electric near-field while also supporting unidirectional forward scattering. Finally, we analyzed the hybrid dimer with substrates of different materials. It supported strong electric high-order moments along the z-axis and x-axis in gaps between MDM nanoparticles and between MDM nanoparticles and the Ge substrate, owing to the intense displacement currents inside of the dielectric layer. We found that the local electric field of this MDM hybrid dimer nanoantenna with Ge substrate was well improved and attained 3325 v/m.

## 1. Introduction

At subwavelength scales, nanoscale structures are critical components for influencing and engineering light’s electromagnetic behavior [1,2]. Plasmonic dimers have received much attention due to their special optical properties in the optical response that restrict the collective oscillation behavior of free electrons near the metal surface at wavelengths ranging from ultraviolet to near-infrared (NIR), a phenomenon known as localized surface plasmon resonances (LSPR) [3]. The electromagnetic field around plasmonic dimers can be enhanced by exciting LSPR modes [4]. Furthermore, by altering the gap width of the dimer, the plasmonic dimer nanoantenna can change the resonance wavelength, providing for great tunability across a large optical spectral range. However, because the induced hybrid electric moments in the gap region maintain a powerful field enhancement “hot-spot”, large absorption loss has been observed to impact plasmonic excitations. This increases the likelihood of nonradiative photon recombination, which may be undesirable for applications such as light radiation [5,6]. This unavoidable problem has prompted researchers to investigate high-permittivity, low-loss dielectric counterparts. A dielectric dimer assists in managing the near-field amplification and far-field radiation properties of exciting light by creating a displacement current inside the nanostructure [7,8]. Although the dielectric dimer has a lower absorption loss than the metallic dimer, its field enhancement ability is much lower. This limits the dimer nanoantenna’s applications in quantum emitter enhancement [9,10] and Surface Enhanced Raman Scattering (SERS) [11].

Metal-dielectric hybrid dimers can benefit from the features of both metal and dielectric materials. Multilayered hybrid nanoantennas can achieve minimal backscattering and unidirectional needle-like radiation at different excitation sources (such as a plane wave or a dipole emitter, respectively) [12,13]. In addition, it is possible to design a multilayer nanoantenna and put it in the experiment with the advancement of nanoparticle manufacturing technology [14,15,16] (e.g., the metal-dielectric-metal nanoparticles can be synthesized by a modified coprecipitation method [17]). These hybrid dimers have not only strong electric-field confinement, but also have the low-loss characteristics of dielectric materials [18]. They can provide an extra degree of freedom in light manipulation owing to the support of different coupling modes between LSPR and Mie resonances. Recently, some researchers reported that hybrid dimer nanoantennas caused an incredible reduction of backward scattering by effectively mixing the LSPR and magnetic resonances [19,20]. Dimer nanostructures can offer very sensitive spectroscopy by combining the advantages of powerful, local electric-field enhancement created in the gap. Nonetheless, the hybridization model has only been used to predict the total extinction spectrum of the strong electric-field enhancement in one nanostructure in recent research. Additionally, the current works did not show other different hybridization models simultaneously containing narrow radiation beamwidth and strong electric-field enhancement in one nanostructure with overlapping electric and magnetic high-order resonances of the dimer. Overlapping electromagnetic high-order resonances can significantly decrease the width of a radiation beam and suppress backward scattering [21]. The primary electric and magnetic moments excited by incident light on one part of the hybrid dimer could induce secondary electric and magnetic moments on the other, which would boost the amplitudes and phases of the dimer’s electric and magnetic dipolar and other high-order resonances in the dimer, as well as its local electric-field and scattering properties [22,23,24].

In order to further investigate the mechanism of unidirectional forward scattering and strong electric-field enhancement through the hybrid nanoantenna, in this work, we first show how the asymmetric hybrid material’s dimer exhibits unidirectional forward scattering and local electric-field enhancement under the excitation of plane waves. The multipole decomposition expansion can be used to assess all of the observable features in the spectrum to identify the relative contributions of distinct electric and magnetic multipolar resonances [25,26]. After that, we examine the corresponding electric near-field in the asymmetric dimer gap to disclose how to effectively excite high-order electric moments and superimpose them with magnetic ones. Finally, we analyze the properties of the hybrid dimer with the substrates of different materials under the excitation of plane waves. We found that the electric field of this MDM hybrid dimer nanoantenna with a Ge substrate was well improved and attained 3325 v/m.

## 2. Materials and Methods

We used the (CST MICROWAVE STUDIO) simulation software to calculate the optical properties of the hybrid nanoantenna under a plane wave with different electric polarization directions. Table 1 shows the structural parameters of the dimer and these two MDM nanoparticles. The scattering field patterns of the hybrid dimer in the free space were acquired by capturing the field components in closed-box monitors, and the electric near-field was obtained by E-filed monitors. When the substrate was included, we recorded the near-field components using the monitor with sufficiently large surfaces (15 × 15 μm^2^) 50 nm below the interface. In all the calculations, a perfectly matched layer boundary was used, and the mesh size (mesh type was tetrahedral) in the vicinity of the dimer was set to 0.1 nm (the area in the gap was set for adaptive mesh refinement).

The nanoantenna’s overall scattering field for the plane wave stimulation was performed by the multipole decomposition expansion, according to [27,28]:

(1) $$C_{sca}=\frac{\pi }{k^{2}}\sum_{l=1}^{\infty }\sum_{m=-l}^{l}\left(2l+1)({\left|a_{lm}\right|}^{2}+{\left|b_{lm}\right|}^{2}\right)$$

where $a_{E}(l,m)$ and $b_{M}(l,m)$ are the electric and magnetic multipole coefficients, respectively. They can be expressed as:

(2) $$\begin{array}{cc} a_{E}(l,m) & =\frac{{\left(-i\right)}^{\left(l-1\right)}k^{2}\eta O_{l}m)}{(E_{0}{\left[\pi (2l+1)\right]}^{\left(1/2\right)}}\int exp(-im\phi )\;\{\left[\Psi_{l}(kr)+\Psi_{l}^{\prime \prime }(kr)\right]P_{l}^{m}(cos\theta )\hat{r}\cdot J_{S,j}(r)\\ & +\frac{\Psi_{l}^{\prime }(kr)}{kr}[(\theta )\hat{\theta }\cdot J_{S,j}(r)-i\pi_{lm}(\theta )\hat{\phi }\cdot J_{S,j}(r)]\}d^{3}r \end{array}$$

(3) $$a_{M}(l,m)=\frac{{\left(-i\right)}^{\left(l-1\right)}k^{2}\eta O_{lm})}{(E_{0}{\left[\pi (2l+1)\right]}^{\left(1/2\right)}}\int exp(-im\phi )j_{l}(kr)[i\pi_{lm}(\theta )\hat{\theta }\cdot J_{S,j}(r)+i\tau_{lm}(\theta )\hat{\phi }\cdot J_{S,j}(r))]d^{3}r$$

where $\Psi_{l}(kr)=krj_{l}(kr)$ is the Riccati–Bessel function, $\Psi_{l}^{\prime }(kr)$ and $\Psi_{l}^{\prime \prime }(kr)$ are their first and second derivatives and the other functions $O_{l}m=\frac{1}{{\left[l(l+1)\right]}^{\left(1/2\right)}}{\left[\frac{(2l+1)(l-m)!}{4\pi (l+m)!}\right]}^{\left(1/2\right)}$, $\tau_{l}m(\theta )=\frac{d}{d\theta }P_{l}^{m}(cos\theta )$, $\pi_{{}_{l}m}(\theta )=\frac{m}{\sin\theta }P_{l}^{m}(cos\theta )$ are associated as the Legendre polynomials $P_{l}^{m}$. These coefficients, $a_{E}(l,m)$ and $b_{M}(l,m)$, determine the electric and magnetic multipoles, namely, the dipole, quadrupole, octupole, hexadecapole, dotriacontapole, and so on. The extinction spectrum is largely influenced by these multipole coefficients (e.g., electric dipole (ED), hexadecapole (EH), dotriacontapole, magnetic octupole (MO), hexadecapole (MH)). Other multipole coefficients were consequently ignored since their contributions to scattering in this investigation were minimal. We wanted to construct a nanoantenna with a hybrid dimer that can control unidirectional scattering and simultaneously enhance the local electric field. For illustration purposes, in our research, we used germanium (Ge) as the nanoparticle’s intermediate layer material and silver (Ag) as the innermost and outmost layers of the nanoparticle material. In the work spectra, germanium’s permittivity and energy dissipation loss are both rather low. The optical properties of Ag and Ge were obtained from Palik [29,30].

## 3. Results

**Figure 1:**
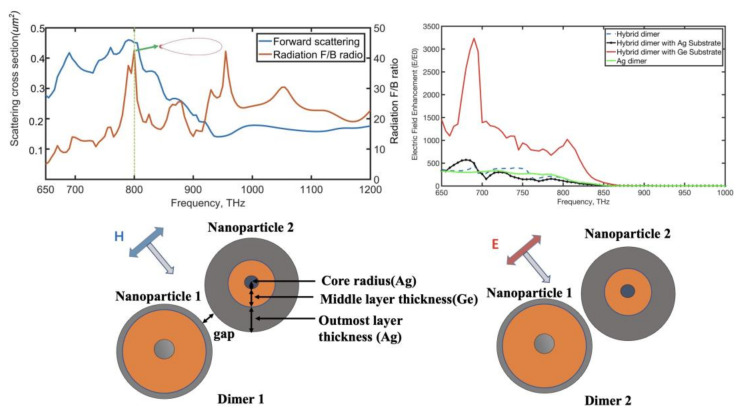
The mechanism of unidirectional forward scattering with narrow radiation beamwidth and strong enhancement of local electric field in the asymmetric metal-dielectric-metal dimer which is excited by incident light with different orientations of electric and magnetic polarization.

**Figure 2:**
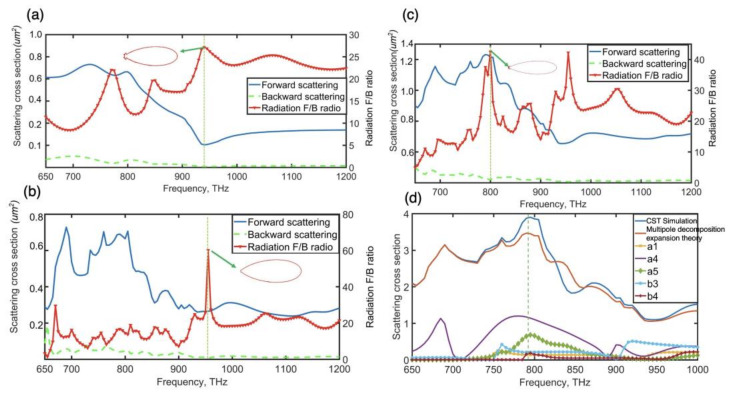
CST simulation structures and results. (**a**–**c**) The scattering field and forward to backward ratio of the large nanosphere (**a**), the small nanosphere (**b**), and the dimer with 40 nm gap (**c**) and theoretical study of coupling situation of scattering spectra is compared to CST simulation results (**d**) and insets displaying radiation pattern diagrams at the specific frequency.

The hybrid nanoantenna was able to control the optical scattering direction and confine the local electric field. When the dimer was excited by the incident wave with various electric polarization orientations, Figure 1 shows how the asymmetric dimer supported unidirectional scattering or a substantial improvement in the electric near-field. We started using the hybrid dimer nanoantenna by searching for a Kerker condition and unidirectional forward scattering. As shown in Figure 2, we initially evaluated the properties of the nanoantenna made up of two different nanospheres operating under a plane wave (the direction of electric polarization was along the y-axis). The dimer contained two MDM nanospheres, of which the structure parameters are shown in Table 1 (dimer 1 gap width = 40 nm). When the individual hybrid nanosphere was excited by a plane wave, it was able to sustain various electromagnetic dipolar or multipolar responses due to induced strong displacement currents in the high-refractive index layer. The hybrid electromagnetic multiple-coupled modes effectively superimposed in the dimer gap made the asymmetric nanoantenna show new features such as unidirectional forward scattering with narrow beamwidth.

For single nanoparticles and dimer configurations, Figure 2 illustrates numerical results of the forward and backward scattering spectra, as well as their F/B ratio. Several peaks were found in the forward to backward ratio spectra of such individual nanospheres at multiple frequencies. Unfortunately, the scattering efficiency at such frequencies was low due to mismatched resonance peaks when the hybrid dimer was excited by the plane wave (the direction of electric polarization was along the y-axis). According to new induced hybrid electromagnetic modes in the gap of the hybrid dimer, the hybrid dimer’s F/B ratio spectra displayed a new unique peak at around 790 THz, where forward scattering reached a maximum value and backward scattering was reduced. These nanoantenna primary lobes’ equivalent beamwidths at the frequencies of highest forward to backward ratios were 40.9 degrees, 40.3 degrees and 30.6 degrees, as shown in Figure 2. In comparison to these two individual particles, the hybrid dimer had the narrowest beamwidth of the major lobes at 790 THz. The induced high-order magnetic resonance in the dimer may have assisted to increase directivity and narrow the radiation beam. The cross-section of scattered-field radiation patterns at the frequencies of maximal F/B ratios is also shown in Figure 2a–c. The scattering strength of the dimer was more than 5 times that of the individual nanoparticle at 790 THz. The asymmetric MDM dimer’s ability to completely utilize the scattering electric and magnetic high-order resonances in the dimer for unidirectional forward scattering was demonstrated by the increase in radiation directivity and scattering intensity.

The CST simulation results regarding the scattering spectrum of the hybrid dimer and the theoretical multipole decomposition findings for partial electric and magnetic multipoles were shown in Figure 2d. At 790 THz, the electric dotriacontapole mode, as well as the magnetic octupole and hexadecapole, clearly dominated the asymmetric hybrid dimer. The electric dotriacontapole mode dominated the smaller MDM particle at about 790 THz, whereas the bigger particle exhibited distinct EO, EH and MH modes. Due to the low and equal intensity of the high-order electric and magnetic modes in single nanopartices, these individual particles displayed the highest radiation F/B ratio at 950 THz, as shown in Figure 2a,b. However, the scattering efficiency at this frequency was low. We created a design that aligns the metal, electric dotriacontapole mode in tiny particles with the dielectric, magnetic hexadecapole mode in big particles at the same frequency of ~790 THz. The electric and magnetic resonances interacted when two MDM nanoparticles were brought together to create a dimer, distorting and creating additional spectrum characteristics. These novel electric and magnetic high-order modes in this hybrid dimer enabled substantial scattering intensity and increased directivity at 790 THz when compared to individual MDM nanoparticles. Therefore, weak magnetic multipolar resonance is considerably boosted and red-shifted to match the electric multipolar resonance in the dimer due to interparticle interaction. This might lead to a near-ideal Kerker situation in the frequency range of 790 THz, close to the dimer’s resonance peak, greatly enhancing scattering efficiency.

**Figure 3:**
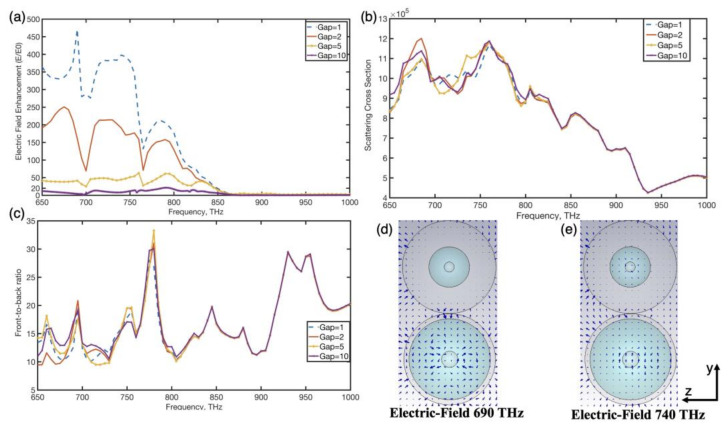
Electric near-field enhancement (**a**), scattering cross-section (**b**) and front-to-back scattering ratio (**c**) of the hybrid dimer for different separating distances in the gaps as a function of frequency. Additionally, inset distributions of (**d**,**e**) show absolute value of the electric field (gap width = 1 nm) at the frequencies of 690 THz and 740 THz, respectively.

When plasmon resonances are generated in the vicinity of metallic nanoparticles, electric-field distributions are also formed outside of the nanoparticle. Similarly, when light excites dielectric nanoparticles, the electromagnetic field is substantially concentrated inside the material due to the creation of powerful displacement currents [31]. The hybrid dimer nanoantenna not only has abilities to control the unidirectional radiation of the light, but also to keep an intensive electric field in the gap. In addition, the asymmetric hybrid nanoantenna showed large electric-field enhancement under the special incident light’s propagation direction along the z-axis in the negative direction (electric polarization parallel to the dimer axis). As a result, as shown in Figure 3, we investigated the hybrid antenna’s electromagnetic properties, referring to a dimer of subwavelength nanoparticles that are close together. The size and material composition of each nanoparticle were shown in Figure 1.

In this study, in order to obtain high electric near-field enhancement, we set the narrow gap width of the coupled antenna as 1 nm (dimer 2: gap width = 1 nm). When the distance between metal surfaces is less than 1 nm, the quantum tunneling effect occurs in the gap. The gap width defines the distance between adjacent spherical surfaces. Figure 3 shows the electric near-field enhancement, scattering cross-section and front-to-back scattering ratio of the hybrid dimer for different separating distances in the gaps as a function of frequency. As shown in Figure 3a, the hybrid dimer showed distinct peaks in its electric near-field enhancement spectra at 690 THz (above 460 V/m) and 740 THz (above 400 V/m). In addition, the electric enhancement coefficient of the hybrid system reduced as the gap width increased. Figure 3d,e show the magnitude distribution of the electric field inside and in the vicinity of the asymmetric dimer at 690 THz and 740 THz, respectively. Unlike pure metals, the applied electric field forms a depolarizing field inside the MDM nanoparticle’s dielectric layer. When the dipolar resonance occurs, this depolarizing field causes intense displacement currents inside of the dielectric layer which can cause an intense dipolar electric near-field around the nanoparticle [32]. When such hybrid nanoparticles constitute an asymmetric dimer nanoantenna, the powerful electric near-field in the gap is caused by the dipolar field distribution combined with nonradiative high-order modes in the dimer. As shown in Figure 3d,e, the high-order resonance mode induced in the dimer dielectric layer effectively overlapped the LSPR in the dimer metal layer, resulting in a strong electric field in the gap. Furthermore, as magnitudes of high-order induced in the dimer dielectric layer decreased, correspondingly, the intensity of the electric near-field in the gap reduced quickly. The hybrid antenna’s far-field features were also investigated. According to Figure 3b, when the hybrid dimer had different gap widths, two obvious scattering peaks were observed at around 690 THz and 760 THz. As shown in Figure 3c, the hybrid dimer showed several peaks in its front-to-back ratio at around 690 THz, 780 THz and 930 THz, at which these forward scatterings attained peaks and backward scattering was suppressed. Compared to a pure material nanoparticle, it was obviously illustrated that the hybrid dimer showed a strong electric near-field (above 460 V/m) and high unidirectional forward scattering peak (F/B surpass 22) at 690 THz, owing to the high-order resonance mode induced in the dimer dielectric layer effectively overlapping the LSPR in the dimer metal layer, which resulted in a strong electric field in the gap.

**Figure 4:**
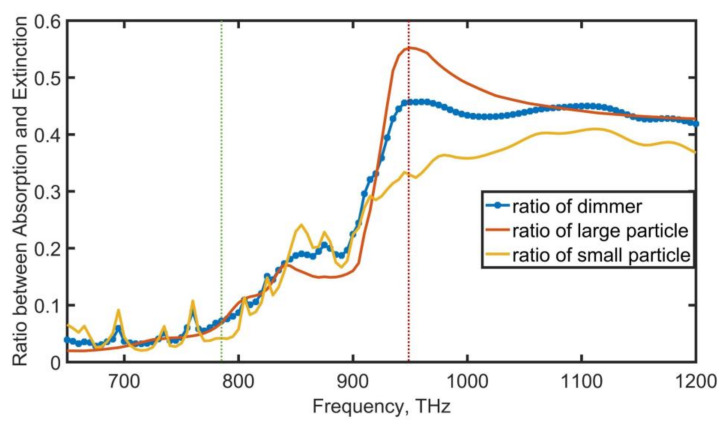
The absorption-to-extinction ratio ($\sigma_{abs}/\sigma_{ext}$) of two individual MDM nanoparticles and MDM hybrid dimer.

Using metal nanoparticles resulted in significant power absorption and energy loss at optical frequencies, which seems unfavorable at first glance. When a metal’s valence electron absorbs a photon and jumps to the Fermi surface or when a photon is absorbed by an electron at the Fermi surface and moves to the next unoccupied state in the conduction band, metal losses arise as a result of interband transitions [33,34]. The absorption-to-extinction (Abs/Ext) ratio ($\sigma_{abs}/\sigma_{ext}$) of these two single MDM nanoparticles and the dimer were shown in Figure 4. The Abs/Ext ratio ($\sigma_{abs}/\sigma_{ext}$) of the individual large and small MDM nanoparticle at the frequency of the maximum radiation F/B ratio (red-dashed) exceeded 30% and 55%, respectively. However, by comparing the Abs/Ext ratio ($\sigma_{abs}/\sigma_{ext}$) of its individual MDM particle, we found that the Abs/Ext ($\sigma_{abs}/\sigma_{ext}$) of this MDM dimer nanoantenna at the frequency of the maximum radiation F/B ratio (green-dashed), $\sigma_{abs}/\sigma_{ext}$ was effectively suppressed under 10%.

**Figure 5:**
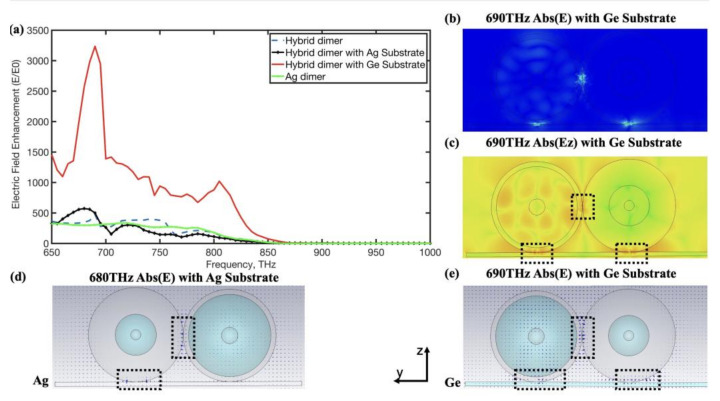
(**a**) The electric near-field enhancement of the hybrid dimer and Ag dimer without substrate and hybrid dimer with Ag/Ge substrate; (**b**,**c**,**e**) distribution of the absolute value of the hybrid dimer with Ge substrate electric field at the frequency 690 THz, and (**d**) the electric field of the hybrid dimer with Ag substrate at 680 THz.

The bridged nanoantenna, which contained about ten nanometers of thick dielectric bridged nanostructure, can enhance an electric near-field by exciting an incomplete quadrupole resonance [31]. Thus, we built an analogous bridged nanoantenna by placing the hybrid dimer on several ten-nanometer thick dielectric substrates. Compared to the hybrid dimer in the air alone, it can improve the electric near-field by placing the hybrid dimer on a metal/dielectric substrate. We chose Ag substrate and Ge substrate with 20 nm thickness. Additionally, these nanodimers were excited by the plane wave propagation direction along the z-axis in the negative direction (electric polarization direction along the y-axis). As shown in Figure 5, we compared the near-field enhancement of the electric field of the hybrid dimer without substrate, the hybrid dimer with Ag substrate, the hybrid dimer with Ge substrate (red line), and the Ag dimer without substrate, showing how the hybrid dimer with metal/dielectric substrate can effectively enhance the local electric field. Compared to other situations, we observed that more than 3325 times the electric-field enhancement was attained around 690 THz when the hybrid dimer was placed on a Ge substrate, as shown in Figure 5a. This is an interesting result, showing that the peak enhancement factors (E2E02)of the hybrid nanoantenna were larger than 10^7^ and may be quoted as surface-enhanced Raman scattering (SERS) enhancements. The SERS factor is proportional to the peak enhancement factors (E2E02). We also investigated the distribution of the absolute value of the hybrid dimer with a Ge substrate electric field at the frequency 690 THz, as shown in Figure 5b. the phenomenon of strong electric-field enhancement was observed simultaneously in the gap between MDM nanoparticles and the gap between MDM nanoparticles and Ge substrate. Unlike individual hybrid dimers and hybrid dimers with Ag substrate, these strong electric-field areas were mostly found in gaps between MDM nanoparticles and dielectric substrates, which depend on induced strong electric high-order moments along the y-axis and the z-axis in gaps. On the contrary, the hybrid dimer with Ag substrate only induced electric high-order moments along the y-axis in gaps, resulting in a less strong electric field in the gap between the MDM nanoparticle and Ag substrate. The hybrid dimer with Ge substrate shows 3325 times electric-field enhancement around 690 THz due to simultaneously effective inducing electric high-order moments along the y-axis and the z-axis. Compared to other conditions, the hybrid dimer with Ge substrate showed large values of E_z_ in gaps between MDM nanoparticles and gaps between MDM nanoparticles and the Ge substrate, as shown in Figure 5c.

We analyzed the hybrid dimer with different material substrates which showed new features. The hybrid dimer with different material substrates formed new nanostructures called bridged nanoantennas. The Ag substrate boosted the coupling of the electric modes between the metal layer and transformed peak enhancement factors of the dimer to the blue spectrum owing to new features of electric modes along the y-axis supported by the close dimer on the Ag substrate. When the hybrid dimer was placed on a Ge dielectric substrate, we observed intense displacement currents inside of the dielectric layer that resulted in strong electric high-order moments along the y-axis and z-axis in gaps between MDM nanoparticles and the Ge substrate. These induced electric high-order moments along the y-axis and z-axis in gaps correspondingly caused a strong electric near- field in these gaps, as shown in Figure 5c,e.

## 4. Conclusions

In conclusion, we theoretically and numerically showed all the optical properties of the hybrid dimer nanoantenna through CST Microwave Simulation software. Under the excitation of a specific light wave at optical frequency, we unequivocally established the mechanism of unidirectional forward scattering and significant electric-field augmentation through the asymmetric dimer. The interparticle coupling and weak magnetic multipolar resonance were significantly enhanced and red-shifted to match the electric multipolar resonance in the dimer, resulting in achieving the Kerker condition around the resonance peak frequency of the dimer. By physically and delicately adjusting the gap spacing sizes, the optimized asymmetric hybrid dimer manifested a high forward-to-backward scattering ratio (surpass 40) with narrow beamwidth (=30.9 deg) and large forward scattering intensity (up to 5 times larger than the single hybrid nanoparticle). To assess the relative contributions of different electric and magnetic multipolar resonances, the multipole decomposition expansion was employed to examine the dimer’s spectrum responses. Furthermore, when the asymmetric hybrid dimer nanoantenna was excited by the incident light (electric polarization direction parallel to the dimer axis), it sustained unidirectional forward scattering and supported the strong electric field around the gap between nanospheres. In addition, we analyzed the hybrid dimer with different material substrates. We found that the electric field of this hybrid dimer nanoantenna with Ge substrate was well improved and attained 3325 v/m. It provided a preliminary assessment of the hybrid MDM dimer antenna’s potential for light directivity modulation and SERS.

## Figures and Tables

**Table 1:** Parameters of the components of the dimer.

Parameters	Core Radius	Middle Layer Thickness	Outermost Layer Thickness	Gap Width
Nanoparticle 1	40 nm	160 nm	25 nm	/
Nanoparticle 2	25 nm	75 nm	130 nm	/
Dimer 1	/	/	/	40 nm
Dimer 2	/	/	/	1 nm

## Data Availability

Not applicable.
